# First person – Tim Petzold

**DOI:** 10.1242/bio.062142

**Published:** 2025-08-14

## Abstract

First Person is a series of interviews with the first authors of a selection of papers published in Biology Open, helping researchers promote themselves alongside their papers. Tim Petzold is first author on ‘ Connexin 41.8 governs timely haematopoietic stem and progenitor cell specification’, published in BiO. Tim conducted the research described in this article while a PhD student in Julien Bertrand's lab at the Department of Pathology and Immunology, Faculty of Medicine, University of Geneva, Switzerland. He is now a postdoc in the lab of Holger Gerhardt at the Max Delbrück Center for Molecular Medicine in the Helmholtz Association, Berlin, Germany, investigating developmental biology – previously his focus was on how blood stem cells develop and now it has shifted to how the vascular system develops.


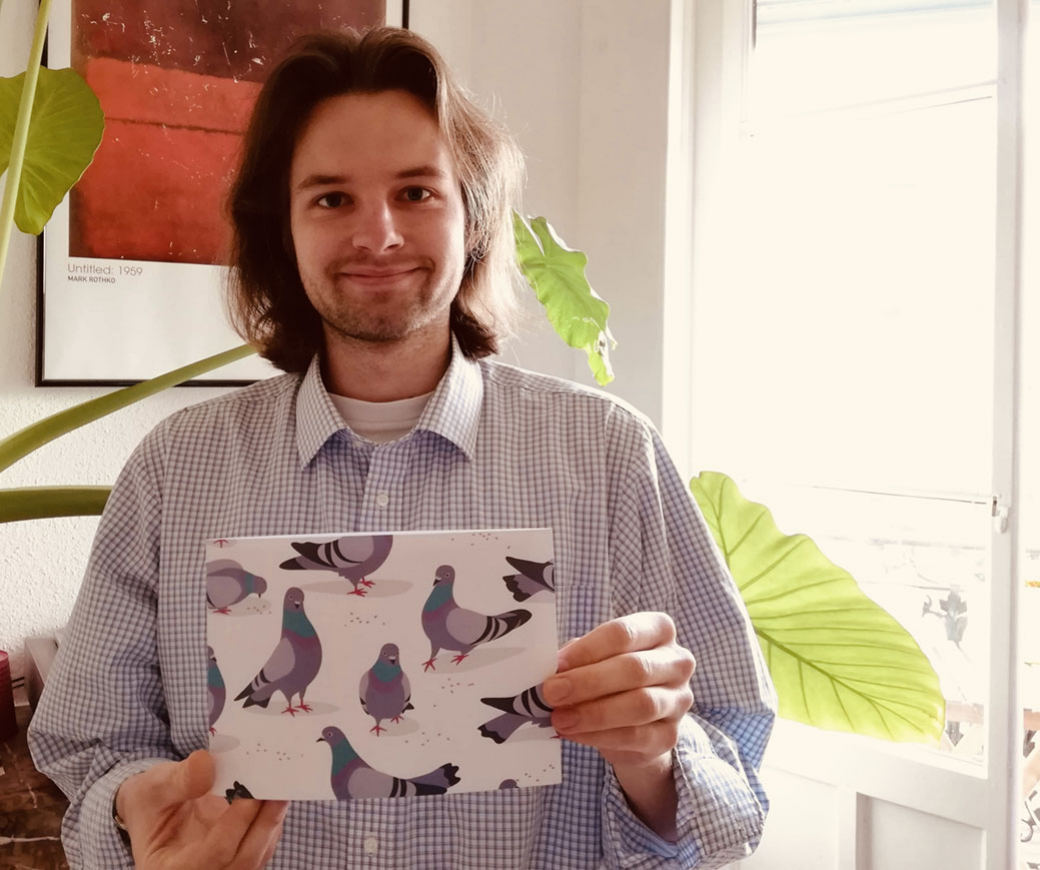


**Tim Petzold**

**Describe your scientific journey and your current research focus**

After working in a number of different fields, including neurobiology, metabolism and epigenetics, I decided to do a PhD in developmental biology, which I did in Julien Bertrand's lab at The University of Geneva. In Julien's lab, I worked on numerous aspects of haematopoietic stem cell development, with a focus on mechanisms governing the specification and expansion of these cells during embryogenesis. Since blood stem cells originate from the vascular endothelial cells and interact with the vasculature during their expansion phase, I became increasingly interested in the vascular development. Hence, early last year, I joined Holger Gerhardt's lab at the Max Delbrück Center in Berlin as a postdoc. In Holger's lab, I am investigating endothelial cell specification and arteriovenous vessel development using the zebrafish embryo as a model system.

**Who or what inspired you to become a scientist?**

As a child, I watched a lot of nature documentaries. Therefore, I was pretty ecstatic to receive a birthday card from David Attenborough himself a few years ago (see photo)! It's a story for another day, but I have to say that sadly I don't know him personally!

During high school, I had a brilliant biology teacher, Brian Marriott. Brian made biology lessons very captivating. I remember vividly how he would get students to demonstrate how the electron transport chain works in what can only be described as a blend of biology, theatre and comedy!

Other than Brian, Matthew Ronshaugen, my undergraduate tutor at The University of Manchester, and Leonie Ringrose, at The Humboldt University in Berlin, both also had a profound impact on my scientific development. Matt and Leonie loved to discuss science with great enthusiasm. It was also clear that they were very passionate about educating the next generation of early-career researchers and enjoyed watching them grow, scientifically.

**How would you explain the main finding of your paper?**

Haematopoietic (blood) stem and progenitor cells can give rise to all other cell types in the blood system. How blood stem and progenitor cells are formed is not completely understood. Previously, we found that zebrafish embryos lacking a gap junction protein named Connexin 41.8, have defective blood stem and progenitor cell proliferation (Cacialli et al., 2021). In this study, we show that when Connexin 41.8 is present but not fully functional, there is a delay in the initial formation of these cells. We then set out to understand why this is, mechanistically. Ultimately, we discovered that Connexin 41.8 plays an important role in switching on a molecular signalling pathway at a very precise moment. This signalling pathway is necessary for the formation of blood stem and progenitor cells at the correct time during the development of the zebrafish embryo. Since mammals also have Connexin 41.8 (named Connexin 40), we speculate that the molecular mechanism we've discovered in this work might also be important for the formation of blood stem and progenitor cells in mammals, including humans.

**Figure:**
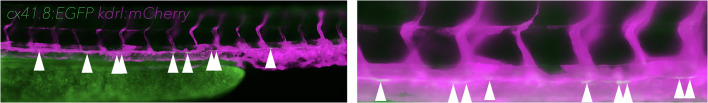
**A wild-type zebrafish embryo trunk at 48 hours post-fertilisation.** Blood vessels are labelled in magenta and white arrowheads point to cells in the floor of the dorsal aorta in which the *connexin 41.8* promoter is active and that will become blood stem cells.

**What are the potential implications of this finding for your field of research?**

A major challenge in regenerative medicine is the *in vitro* generation of haematopoietic stem and progenitor cells, which could, for example, help generate healthy stem cells for patients with blood cancers. We hope that our paper contributes a small piece to the very big puzzle that is understanding all factors required for the initiation of the blood stem and progenitor cell program. As such, our findings will hopefully not only advance knowledge in the field of developmental biology, but also contribute to the enhancement of current regenerative medicine protocols, which may help patients with diseases such as leukaemia in the future.

…this project taught me to keep looking forward and that persistence can eventually pay off

**Which part of this research project was the most rewarding?**

What was most rewarding for me was how this research project came together during the final year of my PhD. This project had taken us in many directions over a number of years without much progress. However, things began to fall into place once we discovered that treating *cx41.8^tq/tq^* mutant embryos with hydrogen peroxide to induce reactive oxygen species production rescued the blood stem and progenitor cell specification defect. Therefore, this project taught me to keep looking forward and that persistence can eventually pay off.

**What do you enjoy most about being an early-career researcher?**

I enjoy many parts of research, so it is difficult to pinpoint which is the most important for me! Something I have always enjoyed is the puzzle-solving involved, for example when trying to disentangle a molecular mechanism. I also enjoy building new tools, such as generating new zebrafish lines, which is something I did a lot of during my PhD. During the first phase of my postdoc, I have also realised just how engaging and joyful it is to work very closely with others on highly collaborative projects. Finally, for me, the process of embryogenesis must be one of the most beautiful things to occur in nature – being able to study it, even just a small aspect of it, is something I'm very grateful for.

**What's next for you?**

I recently joined Holger Gerhardt's lab at the Max Delbrück Center in Berlin as a postdoc. In Holger's lab we work on a wide variety of projects related to vascular biology in development, health, and disease, using both *in vitro* and *in vivo* model systems. I am particularly interested in how endothelial cell fate is induced and how blood vessels are formed. Holger and I wrote an “In preprints” piece for Development on this topic last year (Petzold and Gerhardt, 2024). Currently, I am working on a highly collaborative project, in which we are investigating endothelial cell specification and vascular development during early zebrafish development, using lineage-tracing and cell tracking technologies. I'm really enjoying using new tools and technologies to continue to try to find answers to fundamental developmental biology questions.
